# Autoimmune reactivity to malondialdehyde adducts in systemic lupus erythematosus is associated with disease activity and nephritis

**DOI:** 10.1186/s13075-018-1530-2

**Published:** 2018-02-26

**Authors:** Uta Hardt, Anders Larsson, Iva Gunnarsson, Robert M. Clancy, Michelle Petri, Jill P. Buyon, Gregg J. Silverman, Elisabet Svenungsson, Caroline Grönwall

**Affiliations:** 1 Department of Medicine, Rheumatology Unit, Karolinska Institutet and Karolinska University Hospital, Center for Molecular Medicine, Stockholm, Sweden; 2 Department of Medical Sciences, Clinical Chemistry, Uppsala University, Uppsala, Sweden; 3 Department of Medicine, Division of Rheumatology, NYU School of Medicine, New York, NY USA; 4 Department of Rheumatology, Johns Hopkins University School of Medicine, Baltimore, MD USA

**Keywords:** Autoantibodies, Natural antibodies, Malondialdehyde protein modification, SLE, Lupus nephritis, Disease activity, MDA

## Abstract

**Background:**

Immunoglobulin M (IgM) autoreactivity to malondialdehyde (MDA) protein modifications is part of the natural antibody repertoire in health and may have beneficial functions. In contrast, IgG anti-MDA are increased in chronic inflammation and autoimmunity and may instead have pathogenic properties.

**Methods:**

Herein, we investigated serum IgG anti-MDA levels by enzyme-linked immunosorbent assay (ELISA) in 398 systemic lupus erythematosus (SLE) patients in the Swedish Karolinska SLE cohort and compared these to findings in 225 US SLE patients from New York University and Johns Hopkins University.

**Results:**

In two independent cohorts, IgG anti-MDA levels correlated positively with disease activity by the Systemic Lupus Erythematosus Disease Activity Index (SLEDAI; *p* < 0.0001, Spearman *R* = 0.3). Meta-analysis found an odds ratio of 2.7 (confidence interval (CI) 1.9–3.9; *p* < 0.0001) for high anti-MDA IgG levels with active disease (SLEDAI ≥ 6). Furthermore, IgG anti-MDA correlated directly with erythrocyte sedimentation rate (ESR), C-reactive protein (CRP), soluble tumor necrosis factor receptors (sTNFR-1, sTNFR-2), and vascular cell adhesion molecule 1 (VCAM-1) measurements, and inversely with complement factors (C1q, C2, C3, C4). Importantly, IgG anti-MDA levels were significantly elevated in SLE patients with active nephritis (*p* = 0.0005) and correlated with cystatin C estimated glomerular filtration rate and albuminuria.

**Conclusions:**

Elevated IgG anti-MDA in SLE patients was associated with high disease activity, with active lupus nephritis, and with biomarkers of systemic inflammation. This natural antibody reactivity may have potential prognostic utility, and may also actively contribute to pathogenesis.

**Electronic supplementary material:**

The online version of this article (10.1186/s13075-018-1530-2) contains supplementary material, which is available to authorized users.

## Background

Systemic lupus erythematosus (SLE) is a complex autoimmune disease associated with production of autoantibodies and damage to multiple organs [1]. Lupus nephritis is one of the most serious disease manifestations and is a major cause of morbidity and mortality [2]. There have been reports of a range of different immunoglobulin G (IgG) autoantibody specificities in SLE. However, IgG anti-double-stranded DNA (dsDNA) is one of the most SLE-specific reactivities, present in 40–60% of lupus patients, and though anti-dsDNA antibodies are heterogeneous they are clearly associated with both disease activity and renal disease [3]. In the current study, we investigated autoreactivity to oxidation-associated malondialdehyde (MDA)-modified protein adducts. MDA is generated from lipid peroxidation in states with increased reactive oxygen species (ROS) or during cell death. This highly reactive aldehyde can post-translationally modify proteins through covalent alterations of amino acids carrying amide and amine groups, giving rise to neo-epitopes that elicit antibody responses (reviewed in [4, 5]). Indeed, MDA-modified protein, together with other oxidation-associated modifications, are primary targets recognized by IgM antibodies that are present from birth, also referred to as natural autoantibodies [6–8]. These IgM have been postulated to have roles in maintaining homeostasis, in part by enhancing the clearance of apoptotic cells and harmful modified self-molecules (reviewed in [9, 10]). In fact, raised levels of antibodies to the related but distinct IgM specificity for the phosphorylcholine (PC) head-group in oxidized lipids has been associated with protection from cardiovascular disease and organ damage in SLE [11–15]. In addition, in vitro cell assays and studies in animal models have shown that these types of anti-oxidation neo-epitope-directed IgM can be directly anti-inflammatory [16–18].

In SLE, pathogenic autoantibody production is hypothesized to often be linked to defects in apoptotic cell clearance, which results in immune exposure to intracellular debris, self-antigens, and proinflammatory danger signals [19]. In addition, SLE has been suggested to be associated with a dysregulated metabolic state and elevated levels of ROS, which has been documented by the detection of increased levels of MDA and MDA-modified proteins [20–24]. We hypothesize that uncontrolled ROS and MDA generation may trigger induction of higher levels of anti-MDA antibodies and possibly a class-switch to IgG. Since the IgM anti-MDA antibodies are part of the natural antibody pool, no true breach-of-tolerance event is required and the threshold for the induction of IgG anti-MDA responses may therefore be low. Whereas polymeric IgM antibodies have been associated with protective properties, IgG with the inherent capacity for immune complex formation and engagement of activating Fc-receptors may instead be functionally proinflammatory. However, little is known about IgG anti-MDA autoantibodies and their relationship with biomarkers of clinical disease severity in SLE. In an earlier limited study, we observed increased levels of IgG anti-MDA in SLE patients and an association with disease activity [12]. In the current report, we expanded these studies to a large independent cohort and investigated serum IgG anti-MDA expression and associations with disease activity, renal involvement, and inflammation biomarkers.

## Methods

### Study cohort and clinical assessments

Our cross-sectional study included serum samples from 438 SLE patients fulfilling at least four of the 1982 classification criteria of SLE by the American College of Rheumatology (ACR) [25]. We excluded patients that had received rituximab prior to the visit (*n* = 40). The patient group had a mean age of 47 ± 15 years and 89% of the SLE patient group were female; 38% of the patient group was positive for IgG anti-dsDNA at the time of blood sampling. The disease activity of the patients was assessed by the SLE Disease Activity Index (SLEDAI-2K) [26] and the Systemic Lupus Activity Measure (SLAM) [27]. The SLE-associated organ damage was assessed by the Systemic Lupus International Collaborating Clinics (SLICC)/ACR Damage Index [28]. For evaluation of renal disease activity, the British Isles Lupus Activity Group (BILAG) criteria were used for scoring the activity into grades A–E [29]. Patients with end-stage renal disease at the time of evaluation were excluded from this analysis. Routine laboratory measurements were performed at the Karolinska University Hospital clinical laboratory.

Serum samples were also collected from 322 sex- and age-matched population control subjects, identified in the Swedish national population registry and invited to participate. The only exclusion criterion was a diagnosis of SLE. All study participants were examined by a rheumatologist, and medical records were reviewed.

The Swedish Karolinska SLE cohort was also compared to the US cohort, combining previously collected and reported data from 120 patients from the Johns Hopkins cohort [12] and 105 SLE patients from the New York University (NYU) Langone medical center cohort [13]. The disease activity of the US patients was assessed by the SELENA version of the SLEDAI [30]. For 19 SLE patients in the Johns Hopkins lupus cohort, longitudinal samples were available with 11–20 years between visits. The US SLE samples were compared to nonmatched serum samples collected from 125 healthy blood donors from the blood bank of the Hospital of the University of Pennsylvania and NYU Medical Center.

### Autoantibody assays

Total IgG concentrations were assessed by standard nephelometry at the Karolinska University Hospital clinical laboratory. IgG autoantibody anti-dsDNA, anti-nucleosome, anti-ribosome, anti-cardiolipin (CL), anti-β_2_-glycoprotein-I (β_2_GPI), anti-Smith (Sm), anti-ribonucleoprotein A and 68 (RNP-A, RNP 68), anti-Sjögren’s syndrome antigen A (SSA, anti-Ro52/anti-Ro60), and anti-SSB (anti-La) were analyzed by multiplexed bead technology (Luminex) using the BioPlex 2200 system (Bio-Rad, Hercules) according to the manufacturer’s instructions.

IgG anti-MDA-modified protein adducts and IgG anti-PC were measured by sandwich enzyme-linked immunosorbent assay (ELISA) in the Karolinska SLE cohort as previously described [12]. Briefly, high-binding ELISA plates (Corning) were coated with MDA-modified bovine serum albumin (MDA-BSA; Academy Bio-medical) or PC-BSA conjugate (PC4-BSA; Biosearch Technologies) at 3 μg/ml, and blocked with 3% BSA in phosphate-buffered saline (PBS). Serum was analyzed at 1:200 and 1:1000 dilution and IgG detected with goat F(ab’)_2_ anti-human Fcγ horseradish peroxidase (HRP; Jackson ImmunoResearch). Absorbance at 450 nm was normalized to an internal control sample in relative units (RU)/ml. Repeated freeze-thawing of cryopreserved biobanked serum samples were avoided, although validation studies showed no significant adverse effects on IgG levels after extended storage or freeze-thawing of samples (Additional file 1: Figure S1). IgG anti-MDA -modified protein reactivity in the two SLE cohorts from the US East coast, NYU [7] and Johns Hopkins [12], were previously measured at NYU. The quantitative levels in the Karolinska cohort cannot be directly compared to previously published studies due to changes in the reference sample.

For ELISA competition studies, calf thymus dsDNA (Sigma Aldrich) was captured on microtiter wells coated with activated methylated BSA (Sigma Aldrich). The SLE serum pool was evaluated for reactivity at 1:200 dilution in the presence of soluble antigens at different concentrations. For these studies, MDA-modified BSA was prepared in house. Briefly, MDA was generated by acid hydrolysis of tetramethoxypropane (Sigma Aldrich) and molecular grade BSA (NEB) at 2 mg/ml was modified by 100 mM MDA in PBS (pH 7.4) for 6 h at 37 °C, followed by extensive dialysis to PBS.

### Inflammation biomarkers

High-sensitivity C-reactive protein (hsCRP) and urine albumin were measured with the BN ProSpec System (Dade Behring) and complement factor 3 and 4 (C3 and C4) were analyzed using the IMMAGE™ system (Beckman Coulter). Cystatin C was analyzed on an Architect Ci8200 analyzer with cystatin C reagents from Gentian (Moss). The estimated glomerular filtration rate (eGFR) was calculated from serum cystatin C results in mg/l as previously described [31]. Soluble tumor necrosis factor receptor 1 and 2 (sTNFR-1, sTNFR-2) and vascular cell adhesion molecule 1 (VCAM-1) were analyzed with commercial sandwich ELISA kits (R&D Systems) according to the manufacturer’s instructions. Other variables were determined by routine clinical tests.

### Statistics

Antibody levels were compared using unpaired two-sided Mann-Whitney tests. Spearman correlation was used to analyze associations between variables. Two-sided Fisher’s exact test was used for comparing frequency distributions. Logistic regression models were used for multivariable analysis. The Mantel-Haenszel method with fixed effects was used for meta-analysis combining data from the two cohorts. Analysis was performed using Prism 6 (GraphPad Software Inc.), JMP 13 (SAS Institute Inc.), and Review Manager 5.3 (Copenhagen; The Nordic Cochrane Centre, The Cochrane Collaboration, 2014). A *p* value < 0.05 was considered significant.

## Results

### IgG anti-MDA autoreactive antibodies correlate with disease activity in independent assays and cohorts

**Fig. 1:**
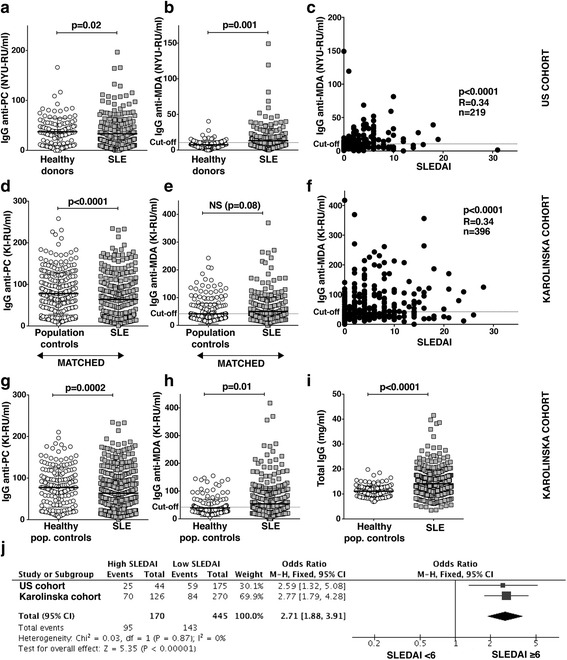
Serum levels of IgG recognizing MDA protein modifications directly correlate with more active SLE in independent cohorts. Serum immunoglobulin G (IgG) levels to malondialdehyde (MDA) protein modification were previously measured by ELISA in 125 healthy blood donors and compared to 120 SLE patients from the John Hopkins cohort [12] and 105 SLE patients from the New York University (NYU) Medical Center cohort [7], here combined and denoted as the US cohort (*n* = 225). In the current study, IgG anti-MDA levels were similarly quantified for 397 SLE patients from the Swedish Karolinska cohort. As a comparison, IgG to another oxidation-associated antigen, phosphorylcholine (PC), was determined in parallel. **a** IgG anti-PC and **b** IgG anti-MDA levels in the US cohort are compared to healthy blood donors. **c** IgG anti-MDA in the US cohort correlates with SLE disease activity by SELENA Systemic Lupus Erythematosus Disease Activity Index (SLEDAI). **d** IgG anti-PC and **e** IgG anti-MDA in 287 SLE patients from the Karolinska cohort compared to sex- and age-matched population controls. **f** IgG anti-MDA serum levels in the Karolinska SLE cohort correlate with SLE disease activity by SLEDAI-2K. **g** Levels of IgG anti-PC,  **h** IgG anti-MDA, and **i** total IgG in 397 SLE patients in the Swedish KI cohort were also compared to the subset of 177 population controls without any chronic diseases, here termed healthy population (pop.) controls, based on medical examination, prescription medications, and self-reported conditions. **j** Meta-analysis of the combined data from the US SLE cohort and the Swedish Karolinska SLE cohort shows a significant association of elevated IgG anti-MDA levels with high disease activity (SLEDAI ≥ 6). Cutoff for high IgG anti-MDA was based on the 75th percentile of the corresponding control cohorts (10.5 NYU-RU/ml for the US cohort or 42 KI-RU/ml for the Karolinska cohort). *P* values are presented from Mann-Whitney analysis (comparing antibody levels in groups), Spearman correlation (correlations of antibody levels with SLEDAI), and the Mantel-Haenszel (M-H) method with fixed effects (meta-analysis). Note that although the assays were similar between the two SLE cohorts, they are not identical and use different relative units, denoted NYU-RU and KI-RU. CI confidence interval, NS not significant, RU relative units

In earlier natural IgM studies [12, 13], we also measured IgG levels to the same oxidation-associated antigens that are recognized by natural IgM. These assays were originally performed as control assays, however we observed an interesting direct correlation between IgG anti-MDA and global disease activity as measured by SLEDAI. Hence, in the present study we further investigated this association of disease activity and clinical manifestation with IgG anti-MDA reactivity in the Karolinska SLE cohort (398 patients). Notably, while we had a striking difference in IgG anti-MDA levels when comparing the combined US SLE cohort (225 patients from NYU and John Hopkins) and the healthy blood donors (13.2 ± 16.4 NYU-RU/ml vs 7.4 ± 5.8 NYU-RU/ml, *p* = 0.001), we did not find the same pattern in the Karolinska SLE cohort compared to the sex- and age-matched population controls (50.3 ± 50.7 KI-RU/ml vs 41.9 ± 37.3 KI-RU/ml, *p* = 0.08) (Fig. 1). The population controls were initially excluded only if they were diagnosed with SLE, and hence other co-morbid conditions could have affected the results. Thus, we repeated the analysis excluding individuals with any chronic condition determined by the rheumatologist’s evaluation, self-reported diseases, and registered prescription medication, resulting in inclusion of 177 healthy population controls. Excluded conditions were, for example, diabetes, psoriasis, renal disease, osteoarthritis, psoriatic arthritis, vitiligo, myasthenia gravis, gout, and cardiovascular disease. Individuals taking medications for chronic pain or inflammation were also excluded even if there were no reported diagnosis. In these comparisons, we then found a distinct difference between population controls and SLE patients that reached statistical significance (*p* = 0.01), although a subgroup of the controls still had substantial anti-MDA levels (Fig. 1). In contrast, IgG anti-PC levels were instead decreased in the Karolinska SLE patients compared to both the matched population controls (*p* = 0.0002) and the healthy population subset controls (*p* = 0.01).

Importantly, in both cohorts we found a significant correlation between levels of MDA reactive autoantibodies and disease activity based on SLEDAI score (US cohort: *R* = 0.34, *p* < 0.0001; Karolinska cohort: *R* = 0.34, *p* < 0.0001; Fig. 1). Furthermore, this correlation was also confirmed in the meta-analysis of the combined data from both studies (Fig. 1 and Table 1), giving an odds ratio (OR) of 2.71 (confidence interval (CI) 1.9–3.9; *p* < 0.0001) for an elevated IgG anti-MDA above cutoff in SLE patients with active disease (SLEDAI ≥ 6). The cutoff for high IgG anti-MDA was defined by the 75th percentile of the corresponding control group. IgG anti-dsDNA positivity, that is included in the SLEDAI score, also showed a strong correlation with high SLEDAI (Table 1 and Additional file 1: Figure S2).

**Table 1:** High IgG anti-MDA serum levels are associated with disease activity in two independent cohorts

	US cohort				Karolinska cohort				Meta-analysis	
	SLEDAI< 6	SLEDAI≥ 6	OR(95% CI)	*p* value^a^	SLEDAI< 6	SLEDAI≥ 6	OR(95% CI)	*p* value^a^	OR(95% CI)	*p* value^b^
IgG anti-dsDNA positive % (*n*/*N*)	47/165	20/28	6.21(2.4–17.5)	**< 0.0001**	73/270	77/126	4.24(2.7–6.7)	**< 0.0001**	4.60(3.1–6.9)	**< 0.0001**
IgG anti-MDA positive % (*n*/*N*)^c^	59/175	25/44	2.59(1.3–5.1)	**0.006**	84/270	70/126	2.77(1.8–4.3)	**< 0.0001**	2.71(1.9–3.9)	**< 0.0001**

The Karolinska cohort used the 2K [26] version and the US cohort used the SELENA [30] version of the Systemic Lupus Erythematosus Disease Activity Index (SLEDAI). Significant *p* values are highlighted in bold

*CI* confidence interval, *dsDNA* double-stranded DNA, *Ig* immunoglobulin, *MDA* malondialdehyde, *n* number of positive patients, *N* total number of patients with available data, *OR* odds ratio

^a^Fisher’s exact test

^b^Mantel-Haenszel fixed effect model

^c^Cutoff for anti-MDA was based on the highest quartile, 75th percentile of corresponding controls, 10.5 NYU-RU/ml for the US cohort and 42 KI-RU/ml for the Swedish Karolinska cohort. Cutoff for IgG anti-dsDNA was according to the clinical assay’s instruction

### IgG anti-MDA reactivity correlates with SLE-associated autoantibodies, but is distinct

**Fig. 2:**
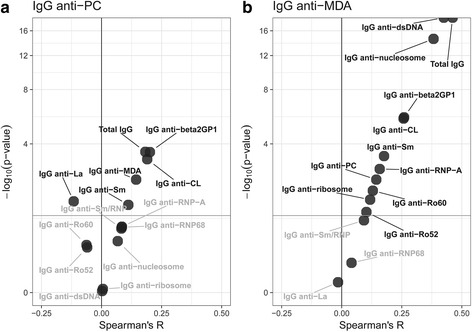
Correlation of oxidation-associated IgG autoantibody reactivity to PC and MDA with lupus-associated autoantibody reactivities. The result from Spearman analysis of the correlation between serum immunoglobulin G (IgG) anti-phosphorylcholine (PC) (**a**) or IgG anti-malondialdehyde (MDA)-modified protein (**b**) levels with SLE-associated IgG autoantibody levels and total IgG in 398 SLE patients from the Karolinska cohort. Spearman *p* values are shown on the *y* axis and Spearman *R* values are shown on the *x* axis for each independent correlation analysis. Significant Spearman correlations with *p* < 0.05 are highlighted in black and nonsignificant correlations are shown in gray. IgG anti-MDA levels correlated especially strongly with IgG anti-nucleosome and IgG anti-dsDNA levels while IgG anti-PC demonstrated a distinct pattern and the strongest correlation with antiphospholipid antibodies (IgG anti-cardiolipin (CL)/β_2_-glycoprotein-I (β_2_GPI)). IgG anti-PC and IgG anti-MDA levels were determined in serum by ELISA. See Additional file 1:Table S1 for more details of the analysis. The figure was generated using the R statistical package (www.r-project.org)

When investigating the correlations between the natural antibody-related IgG specificity for PC or MDA determinants, and SLE-associated IgG autoantibodies, we confirmed that the MDA and PC autoreactivities show distinct correlation patterns. IgG anti-MDA autoantibodies correlated significantly with most of the SLE-associated autoreactivities for membrane-derived, cytosolic and especially nuclear antigens (Fig. 2 and Additional file 1: Table S1 and Figure S3). In contrast, IgG anti-PC did not correlate with any of the most prevalent lupus autoreactivities. Still, IgG anti-PC showed correlation with total IgG and membrane/lipid-associated epitopes (i.e., IgG anti-CL and IgG anti-β_2_GPI; Fig. 2 and Additional file 1: Table S1). For IgG anti-MDA we noted an especially strong correlation with IgG anti-dsDNA levels (*n* = 398, *R* = 0.42, *p* < 0.0001). Similarly, when dichotomizing the patients based on their IgG anti-dsDNA status, IgG anti-MDA levels were significantly higher in the anti-dsDNA-positive patients while there was no difference in IgG anti-PC levels in these two patient subsets (Additional file 1: Figure S4). However, there were certainly patients with elevated IgG anti-MDA levels in the anti-dsDNA-negative group.

MDA adducts and nucleic acid epitopes biologically represent very different types of molecules, but we wondered whether there were polyreactive antibodies capable of binding to both antigen types. However, in ELISA competition assays SLE serum IgG reactivity to surface-bound MDA-modified protein  was only blocked by soluble MDA-modified protein and IgG binding to a dsDNA surface was only blocked by dsDNA in solution (Additional file 1: Figure S3), and there was no significant cross-inhibition. We therefore conclude that these reactivities are concurrent and parallel but the responsible antibodies are not overlapping. Hence, the IgG anti-MDA correlation with SLEDAI cannot be explained by autoantibody cross-reactivity and is not solely due to correlations between antibody tests. Furthermore, to avoid the influence of the total IgG content in individual samples, we normalized the values for specific antibodies for total IgG.

### Levels of IgG anti-MDA correlate with inflammatory biomarkers and low complement

**Fig. 3:**
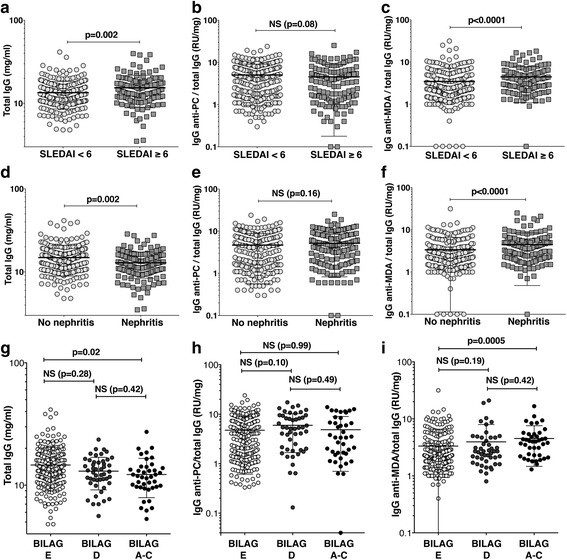
IgG autoreactivity to MDA adducts is elevated in SLE patients with active disease and in patients with lupus nephritis. Levels of immunoglobulin G (IgG) anti-malondialdehyde (MDA) protein adducts in proportion to total IgG were significantly increased in patients with active SLE disease and in patients with nephritis. **a–c** Serum total IgG, and ELISA-determined IgG anti-MDA and IgG anti-phosphorylcholine (PC) levels normalized for total IgG were compared in 270 SLE patients with less disease activity and 126 SLE patients with active disease determined by Systemic Lupus Erythematosus Disease Activity Index (SLEDAI) ≥ 6. **d–f** Total IgG, IgG anti-PC/total IgG, and IgG anti-MDA/total IgG were compared in SLE patients with no history of nephritis (*n* = 241) to patients with nephritis (*n* = 148). The nephritis patient group included all patients with a history of nephritis, independent off status at the time of the visit. **g–i** Total IgG, IgG anti-PC/total IgG, and IgG anti-MDA/total IgG were compared in SLE patients with no history of renal disease (British Isles Lupus Activity Group (BILAG) E; *n* = 228), patients with nephritis in remission (BILAG D; *n* = 48), and patients with active nephritis (BILAG A–C; *n* = 41). *P* values are presented for Mann-Whitney analysis (**a–f**) or Kruskal-Wallis test with Dunn’s correction for multiple comparisons (**g–i**). NS not significant, RU relative units

**Fig. 4:**
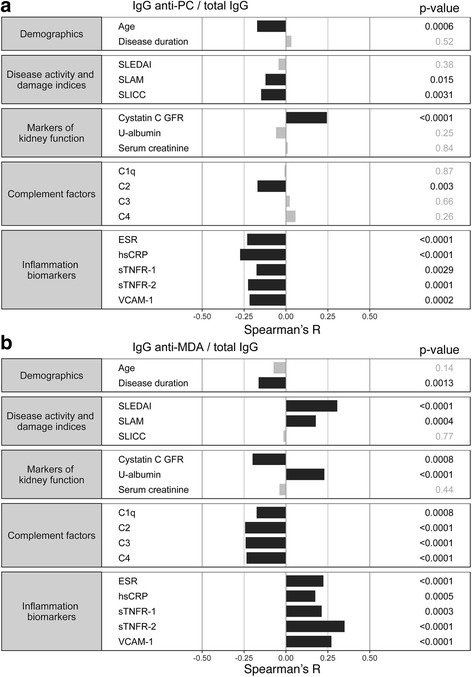
Association of IgG anti-MDA with serological and clinical measurements of disease. The correlation with clinical measurements and biomarkers of immunoglobulin (Ig)G anti-phosphorylcholine (PC) (**a**) and IgG anti-malondialdehyde (MDA) (**b**) normalized for total IgG. Spearman correlation *R* values are shown on the *x* axis. Significant Spearman correlations with *p* < 0.05 are highlighted in black and nonsignificant correlations are shown in gray. Notably, IgG anti-MDA significantly directly correlated with inflammation biomarkers and disease activity, while IgG anti-PC generally demonstrated an inverse correlation with inflammation measurements. IgG anti-MDA also significantly correlated with low complement (C) levels. See Additional file 1:Table S3 for more details of the analysis. The figure was generated using the R statistical package (www.r-project.org). ESR erythrocyte sedimentation rate, GFR glomerular filtration rate, hsCRP high-sensitivity C-reactive protein, SLAM Systemic Lupus Activity Measure, SLEDAI Systemic Lupus Erythematosus Disease Activity Index, SLICC Systemic Lupus International Collaborating Clinics, sTNFR soluble tumor necrosis factor receptor, VCAM-1 vascular cell adhesion molecule 1

When stratifying the patients based on disease activity, with SLEDAI ≥ 6 defined as active disease, the active patient group displayed significantly higher levels of IgG anti-MDA normalized for total IgG (IgG anti-MDA/total IgG) compared with those in the less active group (4.5 ± 3.4 RU/mg vs 3.5 ± 3.6 RU/mg, *p* < 0.0001; Fig. 3). However, this association did not remain significant in logistic multivariate analysis after correction for anti-dsDNA positivity (*p* = 0.20). Indeed, IgG anti-dsDNA normalized for total IgG correlated directly with SLEDAI (Additional file 1: Table S2) and was significantly higher in the high SLEDAI group (4.5 ± 7.5 RU/ml vs 1.2 ± 2.5 RU/ml, *p* < 0.0001; Additional file 1: Figure S5). This was expected, since the score includes an anti-dsDNA serology component. Moreover, the high SLEDAI group also had significantly higher total IgG levels (*p* = 0.002) as well as a trend for a lower IgG anti-PC/total IgG (*p* = 0.08) (Fig. 3). The normalized IgG anti-MDA also correlated with disease activity using SLEDAI [26] (*R* = 0.31, *p* < 0.0001) or the SLAM index [27] (*R* = 0.18, *p* = 0.0004), but did not correlate with organ damage by SLICC [28] (Fig. 4 and Additional file 1: Table S3). In line with these observations, we found an inverse correlation between anti-MDA IgG/total IgG with soluble complement factors C1q (*R* = −0.18, *p* = 0.0008), C2 (*R* = −0.24, *p* < 0.0001), C3 (*R* = −0.24, *p* < 0.0001), and C4 (*R* = −0.24, *p* < 0.0001) (Fig. 4 and Additional file 1: Table S3), suggesting associations with complement consumption via the classical pathway. Notably, low complement levels are also included in the SLEDAI-2K index [26]. In addition, we detected a direct correlation of IgG anti-MDA with the high-sensitivity measurement of C-reactive protein (hsCRP, *R* = 0.22, *p* < 0.0001) and erythrocyte sedimentation rate (ESR; *R* = 0.17, *p* = 0.001) as general measures of acute phase reactants (Fig. 4 and Additional file 1: Table S3). IgG anti-MDA/total IgG displayed a significant direct correlation with serum levels of both VCAM-1 (*R* = 0.27, *p* < 0.0001), sTNFR-1 (*R* = 0.21, *p* = 0.0003), and sTNFR-2 (*R* = 0.35, *p* < 0.0001) (Fig. 4, Additional file 1: Table S3). In addition, in our studies we confirmed that sTNFR-2 significantly correlated with disease activity by both SLEDAI (*R* = 0.31, *p* < 0.0001) and SLAM (*R* = 0.28, *p* < 0.0001) (Additional file 1: Table S4). Intriguingly, in light of the strong correlation with disease activity, we performed a limited study of 19 longitudinal samples stretching over 20 years, and were surprised to document that the IgG anti-MDA levels were relatively stable in many SLE patients over the long term (Additional file 1: Figure S6).

Importantly, after normalization for total IgG, IgG anti-PC and anti-MDA still showed distinct correlation patterns with serological and clinical manifestations. In contrast to anti-MDA reactivity, IgG anti-PC reactivity showed an inverse correlation with disease activity by SLAM (*R* = −0.12, *p* = 0.02), with the acute-phase reactants ESR (*R* = −0.23, *p* < 0.0001) and hsCRP (*R* = −0.27, *p* < 0.0001), as well as with sTNFR-1 (*R* = −0.18, *p* = 0.003), sTNFR-2 (*R* = −0.23, *p* = 0.0001), and VCAM-1 (*R* = −0.22, *p* = 0.0002). In conclusion, while IgG anti-PC may have protective properties, IgG anti-MDA display direct correlations with disease activity , suggesting more potentially pathogenic functionality. In the Karolinska cohort, 32% of the patients were positive for increased IgG anti-MDA/total IgG levels (Additional file 1: Table S5). Consistent with previously observed associations, these patients had increases in disease activity by SLEDAI (OR = 2.65; *p* < 0.0001) and SLAM (OR = 1.56; *p* = 0.04) as well as elevated ESR (*p* = 0.0002). They also had slightly shorter disease duration (*p* = 0.0008). Interestingly, the high anti-MDA patients were found to be more likely to have an antiphospholipid syndrome (APS) autoantibody profile (OR = 1.96; *p* = 0.02) and a history of cardiovascular events (OR = 1.74; *p* = 0.03). We have previously reported that patients with APS autoantibody profile that includes anti-CL/ β_2_GPI represent a distinct lupus subgroup associated with HLA DRB1 *04 and higher natural IgM levels [32]. IgG anti-MDA-positive patients also displayed lower frequency of skin manifestations and photosensitivity. Importantly, we observed higher levels of U-albumin (*p* = 0.0005) and a significantly increased risk of renal disease in IgG anti-MDA-positive patients (OR = 2.1; *p* = 0.001; Additional file 1: Table S5).

### IgG anti-MDA autoreactivity is elevated in SLE patients with renal involvement

In the Karolinska cohort, 148 of 389 SLE patients (38%) with documentation for nephritis status had a history of renal involvement based on ACR criteria [25]. There was no significant difference in age or disease duration between patients with and without nephritis, although some trends for lower age in the nephritis group could be observed (Table 2). However, we found there were relatively more males among the nephritis patients (16.9% vs 8.3%, *p* = 0.01). Importantly, our analysis reveals that SLE patients with a history of, or currently active nephritis, have a significantly increased proportion of IgG anti-MDA among total IgG (i.e. IgG anti-MDA/total IgG), compared to patients without a history of renal disease (4.5 ± 4.0RU/mg vs 3.4 ± 3.3 RU/mg, *p* < 0.0001; Fig. 3 and Table 2). This correlation was maintained in multivariable logistic regression analysis after adjusting for age, sex, disease duration, and serum levels of IgG anti-dsDNA (*p* = 0.04; Table 2). Consistent with the previous literature [33, 34], IgG anti-dsDNA normalized for total IgG levels were significantly higher in the patient group with a history of renal involvement (3.4 ± 6.6 RU/mg vs 1.5 ± 3.5 RU/mg, *p* < 0.0001; Table 2 and Additional file 1: Figure S5). We also performed a more detailed evaluation of the renal status of the patients using renal BILAG scores in a strategy similar to that previously described [35] and we found that IgG anti-MDA/total IgG was significantly increased in patients with current renal activity (BILAG classification A–C, *n* = 41) compared to patients without a history of renal involvement (BILAG classification E, *n* = 228) (4.5 ± 3.1 RU/mg vs 3.3 ± 3.2 RU/mg, *p* = 0.0005; Fig. 2). There also seemed to be a trend for increased levels in patients with previous renal disease (BILAG classification D, *n* = 48; 4.0 ± 4.0 RU/mg), although this was not statistically significant. IgG-normalized IgG anti-MDA but not IgG anti-PC also correlated with eGFR (*R* = 0.24, *p* < 0.0001) as a measurement of impaired kidney function, and albumin in the urine (*R* = 0.23, *p* < 0.0001) (Fig. 4 and Additional file 1: Table S3).

**Table 2:** IgG anti-MDA levels are elevated in patients with nephritis

	No nephritis^a ^*(N = 241)*	Nephritis *(N = 148)*	OR (95% CI)	*p value* ^b^	*Adjusted p value* ^c^
Female, % (*n*/*N*)	92% (221/241)	83% (123/148)	0.45 (0.23–0.83)	**0.01**	*N/A*
Age (years)	47.8 ± 15.4	45.5 ± 14.4	*N/A*	NS (0.12)	*N/A*
Disease duration (years)	12.8 ± 12.2	13.9 ± 11.6	*N/A*	NS (0.18)	*N/A*
ESR (mm/h)	25.4 ± 19.7	27.8 ± 22.8	*N/A*	NS (0.50)	NS (0.34)
hsCRP (μg/ml)	5.0 ± 10.2	4.3 ± 6.4	*N/A*	NS (0.85)	NS (0.74)
Total IgG (mg/ml)	14.9 ± 6.0	12.9 ± 4.5	*N/A*	**< 0.0001**	**< 0.0001**
IgG anti-dsDNA/total IgG (RU/mg)	1.5 ± 3.5	3.4 ± 6.6	*N/A*	**< 0.0001**	*N/A*
IgG anti-PC/total IgG (KI-RU/mg)	4.7 ± 4.1	5.2 ± 4.4	*N/A*	NS (0.16)	NS (0.25)
IgG anti-MDA/total IgG (KI-RU/mg)	3.4 ± 3.3	4.5 ± 4.0	*N/A*	**< 0.0001**	**0.04**
Positive dsDNA IgG (> 10 RU/ml)	31% (74/240)	49% (72/148)	2.1 (1.4–3.2)	**0.0005**	*N/A*
High IgG anti-MDA/total IgG (> 3.8 KI-RU/mg)	26% (62/241)	42% (62/148)	2.1 (1.3–3.2)	**0.001**	**0.02**

Values are shown as mean ± SD or % (*n*/*N*)

*CI* confidence interval, *dsDNA* double-stranded DNA, *ESR* erythrocyte sedimentation rate, *hsCRP* high-sensitivity C-reactive protein, *Ig* immunoglobulin, MDA malondialdehyde, *n* number of positive patients, *N* total number of patients with available data, *N/A* not available, *NS* not significant, *OR* odds ratio, *PC* phosphorylcholine, *RU* relative units Significant *p* values are highlighted in bold

^a^Nephritis was defined according to the 1982 revised ACR criteria, by proteinuria > 5 g per day or greater than 3+ by dipstick if quantification was not performed, and/or cellular casts [25]

^b^*p* value from Mann-Whitney analysis (continuous variables) or Fisher’s exact test (frequencies)

^c^*p* value adjusted for sex, age, disease duration, and IgG anti-dsDNA levels

In summary, MDA-reactive IgG levels were elevated in patients with lupus nephritis and correlated with markers of kidney involvement.

## Discussion

In the current study, we demonstrated that IgG antibodies to oxidation-associated malondialdehyde protein modifications correlate with higher disease activity, active nephritis, and proinflammatory biomarkers in SLE patients. Importantly, we hypothesized that during active autoimmune pathogenesis increases in oxidation-associated tissue injury, and burden of apoptotic cell debris, trigger the elevated expression of IgG anti-MDA antibodies. This in turn may further contribute to pathogenesis by the formation of immune complexes with immunogenic MDA-modified proteins.

Serum IgG anti-MDA are predominantly of the IgG1 and IgG3 subclasses that are complement-fixing and FcγR-activating [12]. This further supports that IgG anti-MDA could contribute to the immune complex deposition and activation of inflammatory pathways, which are hypothesized to be major contributors to the pathogenesis in SLE and particularly to lupus nephritis. Previous reports have shown that circulating MDA-modified proteins are increased in autoimmune disease, presumably as a result of inflammation-altered regulation of oxidation [20, 22–24, 36, 37]. Oxidized low-density lipoprotein (LDL), carrying MDA adducts, has also been shown to be higher in SLE patients and especially in patients with nephritis [21]. While nucleic acid-containing antigens can activate endosomal Toll-like receptors (TLRs) through FcγR internalizations [38], the pathways for MDA-containing antigens may be distinctly different. Interestingly, malondialdehyde and malondialdehyde acetaldehyde (MAA) modifications of amino acids can lead to structural and functional protein alterations that have been reported to be both proinflammatory and immunogenic [39–41].

In our studies, the representation of IgG anti-PC was also investigated. The MDA and PC epitopes both arise as a consequence of oxidative injury, and are present in oxidized LDL (oxLDL) and on apoptotic cells [6]. However, these small epitopes are very different molecular entities that are recognized by different sets of antibodies, and anti-PC antibodies bind to the phospholipid headgroup in oxidatively modified lipids while anti-MDA antibodies recognize MDA carbonylated amino acids in protein antigens. IgM anti-PC and IgM anti-MDA are considered prototypic natural antibody epitopes in murine and human repertoires [6]. Notably, IgM anti-MDA are more dominant in human newborns while IgM anti-PC are low at birth, but highly prevalent in adults and hence must be induced later in life [7]. The PC epitope is also present in cell wall polysaccharide in certain Gram-positive bacteria, and therefore levels of anti-PC antibodies may also to a certain degree be regulated by exposure to microbes. These natural antibodies are associated with the IgM or IgA isotypes and are suggested to often be spontaneously expressed by innate-like B1 cells during T cell-independent responses [42]. Consequently, the immunobiological origins of IgG antibodies to the same epitopes and the relationship to “natural antibodies” has been less clear. However, B1 cells have more recently been reported to interact with antigen-presenting cells [43] and may undergo isotype class switching [44]. Furthermore, anti-MDA and anti-PC expression can to some extent be T cell-dependent [14].

Notably, we observed that levels of IgG anti-MDA strongly correlated with several disease-associated autoantibody types, including anti-dsDNA. However, these antibodies appeared to be completely independent as we could not find any evidence that there would be any cross-reactivity in these binding assays. Similarly, monoclonal anti-MDA antibodies isolated from rheumatoid arthritis (RA) patients [45] neither cross-react with dsDNA in direct binding assays nor display anti-nuclear antibody (ANA) reactivity by Hep2 cell staining (data not shown). The co-existence of the different autoantibody reactivities may instead reflect the immunological profile of certain SLE patients.

Herein, we primarily used the SLEDAI score, which represents an ordinal composite scale for measuring disease activity in SLE patients, for assessing the association of IgG anti-MDA with active disease. Although this scale is  well validated and widely utilized, the heterogeneity of patients and the multiple organ assessment criteria provide challenges for the accurate depiction of SLE disease activity. Thus, several other assessment tools have also been developed [46]. Notably, the SLEDAI, but not the SLAM, incorporates serological measurement of IgG anti-dsDNA and of low complement, which were both shown to correlate with IgG anti-MDA levels in our study. Hence, the stronger correlation of IgG anti-MDA with SLEDAI may in part be a consequence of the integration of serologic components in this index. While we initially hypothesized that IgG anti-MDA levels vary over time due to changes in disease activity and associated pathologic inflammation, our limited longitudinal investigations instead suggested that IgG anti-MDA are relatively stable. However, future extended longitudinal studies are required to fully determine if IgG anti-MDA levels vary over time in association with disease activity and flares, or if these levels remain consistent. Furthermore, it would be of great interest to see if elevation of IgG anti-MDA levels precedes disease onset as reported for many other autoreactivities, or if the levels only increase in a setting of active chronic inflammation. Notably, IgG anti-MDA also correlated with the independent inflammation biomarkers sTNFR-1, sTNFR-2, and VCAM-1 in our studies. sTNFR and circulating VCAM-1 have previously been reported to be increased in SLE in association with flares and higher disease activity [47–49], and sTNFR-2 can be a marker of kidney damage in lupus nephritis [50].

The relationship with the natural antibody subset makes these anti-MDA autoantibodies different from the classical disease-associated IgG, and notably IgG anti-MDA is not specific for SLE. In recent studies, we isolated representative synovial B cell-derived human monoclonal IgG anti-MDA antibodies from RA patients, and these were shown to have potential pathogenic functionality and enhanced osteoclastogenesis in vitro [45]. We also reported elevated levels of IgG anti-MDA in RA patients compared to healthy blood donors and a significant correlation between serum levels and RA disease activity assessed by DAS28 [45].

These previous studies showed low or undetectable levels of IgG anti-MDA in healthy blood donors [12, 45]. However, in the current studies, we observed substantial levels in many individuals in the lupus-unaffected control group. This could be due to differences in the assay between studies, or differences in the immune systems of the control group. The only exclusion criteria for participation in the Karolinska population control group was the SLE diagnosis, and consequently some individuals had other chronic diseases. When we removed all individuals with chronic conditions we observed a decrease in average IgG anti-MDA levels. Hence, the data suggest that the levels of IgG anti-MDA are normally low in healthy individuals, but may be elevated in several chronic diseases.

## Conclusions

While IgG to MDA-modified protein adducts are not specific for SLE, serum levels strongly correlate with lupus disease-associated autoantibody levels. This specificity therefore represents another layer of autoreactivity in SLE and may be part of a polyclonal autoimmune response that plays roles in autoimmune pathogenesis by being the source of proinflammatory immune complexes. Therefore, the routine monitoring of anti-MDA responses may provide additional information about the immunological profile, phenotype, and disease activity in SLE patients. The functionality of IgG immune responses to oxidation-associated protein modifications in SLE merits further studies.

## Additional file

Additional file 1: Additional results and analyses. (PDF 2008 kb)

## Abbreviations

ACR: American College of Rheumatology
APS: Antiphospholipid syndrome
BILAG: British Isles Lupus Activity Group
BSA: Bovine serum albumin
C1q: Complement component 1q
C2: Complement component 2
C3: Complement component 3
C4: Complement component 4
CI: Confidence interval
CL: Cardiolipin
CRP: C-reactive protein
dsDNA: Double-stranded DNA
eGFR: Estimated glomerular filtration rate
ELISA: Enzyme-linked immunosorbent assay
ESR: Erythrocyte sedimentation rate
hsCRP: High-sensitivity C-reactive protein
Ig: Immunoglobulin
LDL: Low-density lipoprotein
MAA: Malondialdehyde acetaldehyde
MDA: Malondialdehyde
NYU: New York University
OR: Odds ratio
PBS: Phosphate-buffered saline
PC: Phosphorylcholine
RA: Rheumatoid arthritis
RNP: Ribonucleoprotein
ROS: Reactive oxygen species
SD: Standard deviation
SLAM: Systemic Lupus Activity Measure
SLE: Systemic lupus erythematosus
SLEDAI: Systemic Lupus Erythematosus Disease Activity Index
SLICC: Systemic Lupus International Collaborating Clinics
Sm: Smith
SSA: Sjögren’s syndrome antigen A
SSB: Sjögren’s syndrome antigen B
sTNFR: Soluble tumor necrosis factor receptor
VCAM-1: Vascular cell adhesion molecule 1
β_2_GPI: β_2_-Glycoprotein-I
