# Supplementary material for: Autoimmune reactivity to malondialdehyde adducts in systemic lupus erythematosus is associated with disease activity and nephritis
Source: Arthritis Res Ther. 2018 Feb 26;20:36. doi: 10.1186/s13075-018-1530-2 (PMC5827973; doi:10.1186/s13075-018-1530-2)
Supplement: Supplementary file 1 — Additional results and analyses. (PDF 2008 kb) [file 13075_2018_1530_MOESM1_ESM.pdf]

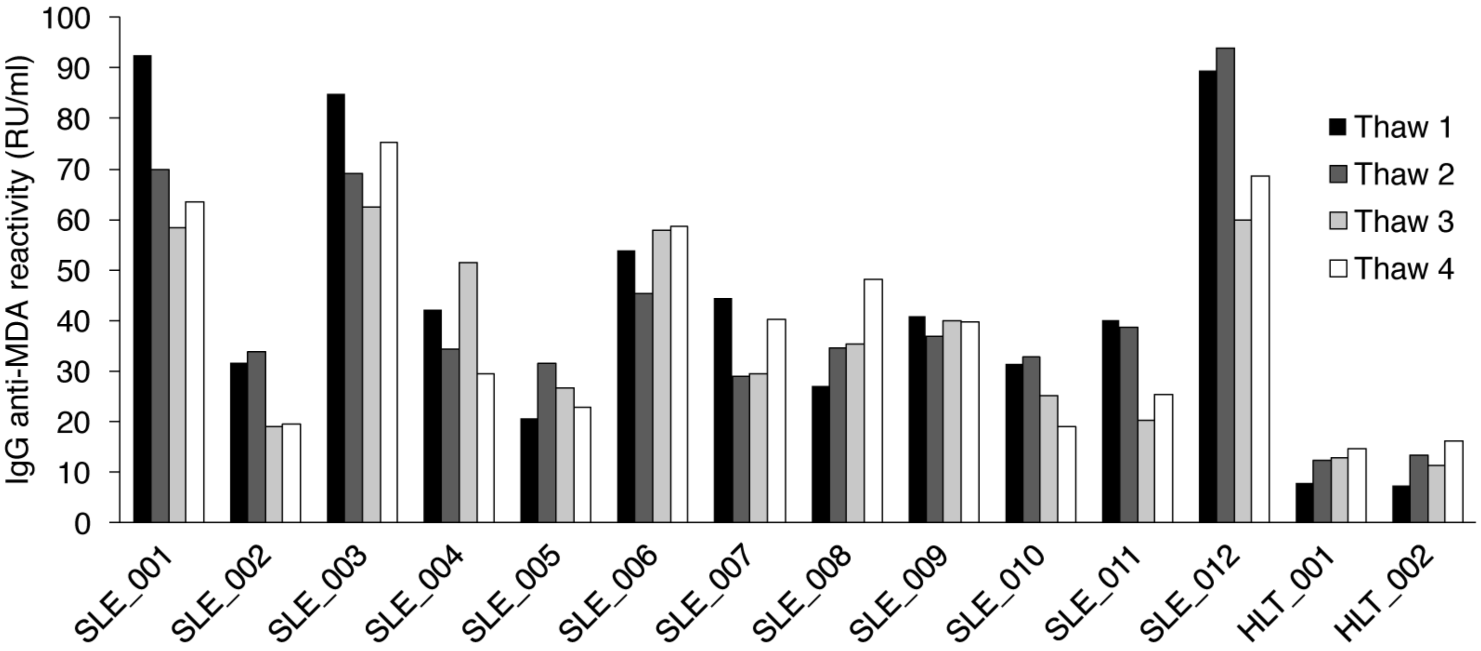

Figure S1. Stability of IgG anti-MDA detection in cryopreserved serum samples

IgG anti-MDA levels were measured in cryopreserved serum samples from 12 SLE patients and two healthy controls after repeated freeze thawing. The IgG levels were not significantly affected by storage or freeze-thawing.

	High SLEDAI		Low SLEDAI			Odds Ratio
Study or Subgroup	Events	Total	Events	Total	Weight	M-H, Fixed, 95% CI
US east coast cohort	20	28	47	165	17.7%	6.28 [2.59, 15.24]
Swedish KI cohort	77	126	73	270	82.3%	4.24 [2.71, 6.64]
**Total (95% CI)**		**154**		**435**	**100.0%**	**4.60 [3.09, 6.86]**
Total events	97		120			

Heterogeneity: $Chi^2 = 0.60$, $df = 1$ ($P = 0.44$); $I^2 = 0\%$

Test for overall effect: $Z = 7.50$ ($P < 0.00001$)

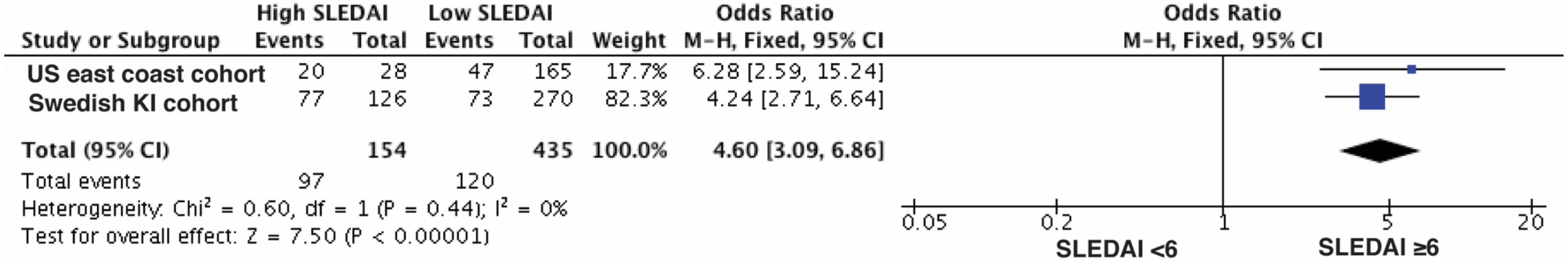

Figure S2. Meta-analysis of IgG anti-dsDNA compared to disease activity in two independent cohorts

Serum IgG anti-dsDNA positivity was determined in 193 SLE patients from the US East coast cohort by ELISA (Inova Diagnostics, Quanta Lite dsDNA) and in 396 SLE patients from the Swedish Karolinska cohort using multiplex bead analysis (BioRad, Bioplex 2200 ANA screen). Cutoff was set according to the manufacturers' instructions. The frequency of dsDNA positivity was compared in patients with active disease, SLEDAI≥6 and patients with less active disease SLEDAI<6 using the Mantel-Haenszel method with fixed effects for meta-analysis.

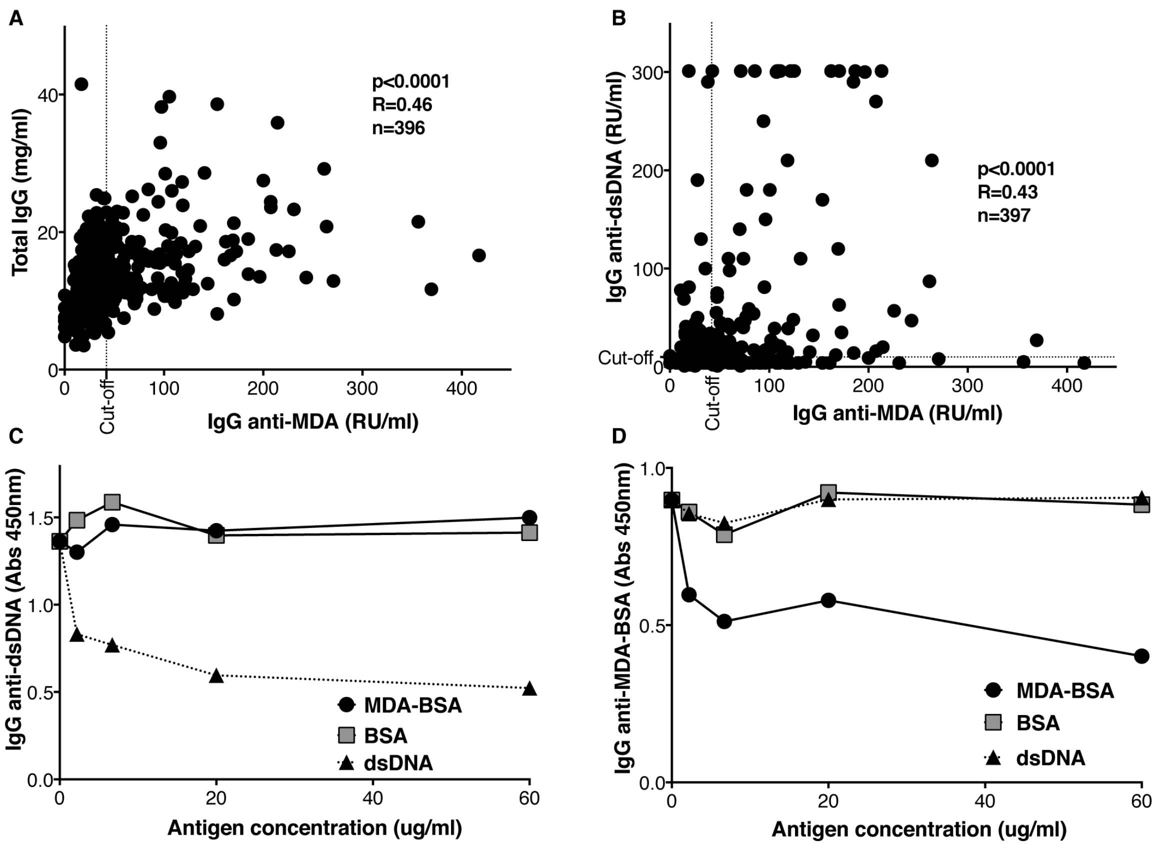

Figure S3. IgG anti-MDA correlates with IgG anti-dsDNA but represents a parallel non-overlapping autoreactivity

Serum levels of IgG anti-MDA modified protein adducts measured by ELISA strongly correlated with total IgG levels (**A**) and levels of IgG anti-dsDNA (**B**) in 397 SLE patients from the Swedish KI cohort. Yet, no cross-reactivity between anti-dsDNA and anti-MDA antibodies was detected in ELISA competition experiments, evaluating binding of IgG in a SLE serum pool at 1:200 dilution to either a dsDNA surface (**C**) or a MDA-modified BSA surface (**D**) in the presence of soluble antigens at indicated concentrations. Only dsDNA in solution could block IgG anti-dsDNA binding and similarly only MDA-BSA in solution could block binding of IgG anti-MDA**.**

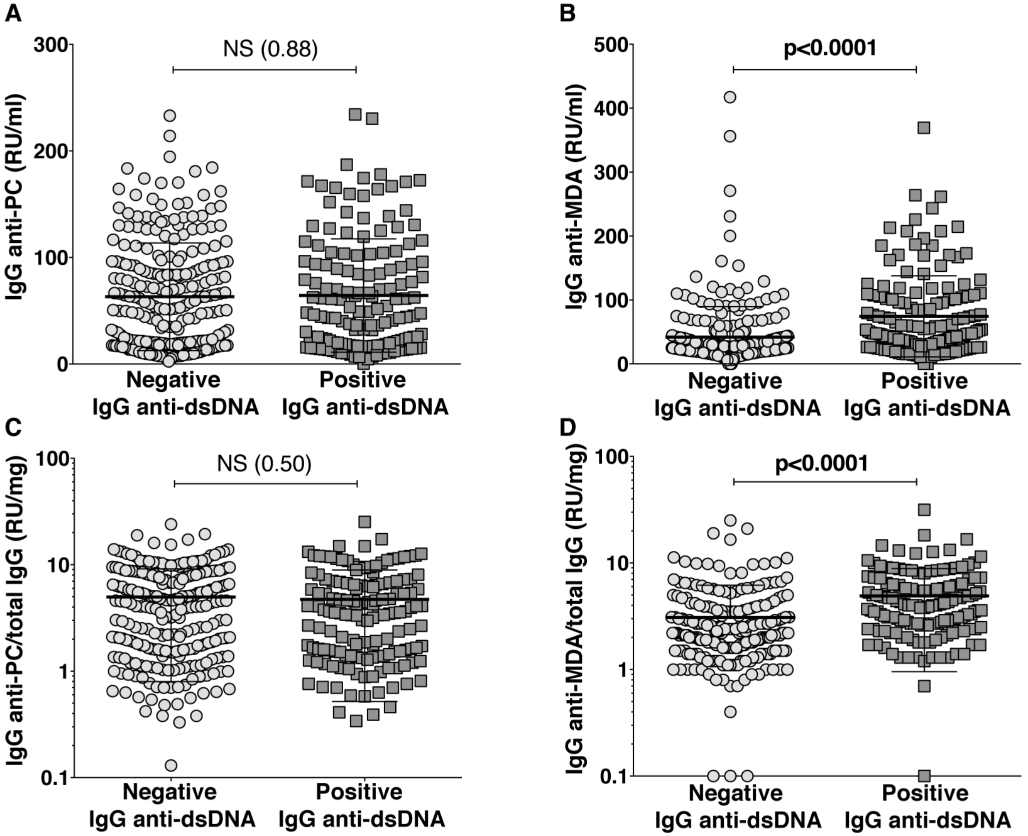

Figure S4. IgG anti-MDA levels are higher in patients with a positive IgG anti-dsDNA test

Serum levels of IgG anti-PC (**A**) and IgG anti-MDA (**B**) were measured by ELISA in 246 anti-dsDNA negative patients and 149 anti-dsDNA positive SLE patients from the Swedish Karolinska cohort. **C-D.** Levels normalized for total IgG. IgG anti-MDA levels were significantly higher in patients with anti-dsDNA compared to patients that were negative (74.5±63 RU/ml vs 41.8±48 RU/ml). The levels of IgG anti-MDA normalized for total IgG were also higher in the anti-dsDNA positive patients (4.9±4 RU/mg vs 3.1±3 RU/mg). No differences were seen for IgG anti-PC levels (64.4±53 RU/ml vs 63.2±51 RU/ml) or IgG anti-PC/total IgG (4.7±4.2 RU/mg vs 5.0±4.2 RU/mg). P-values are presented from Mann-Whitney analysis.

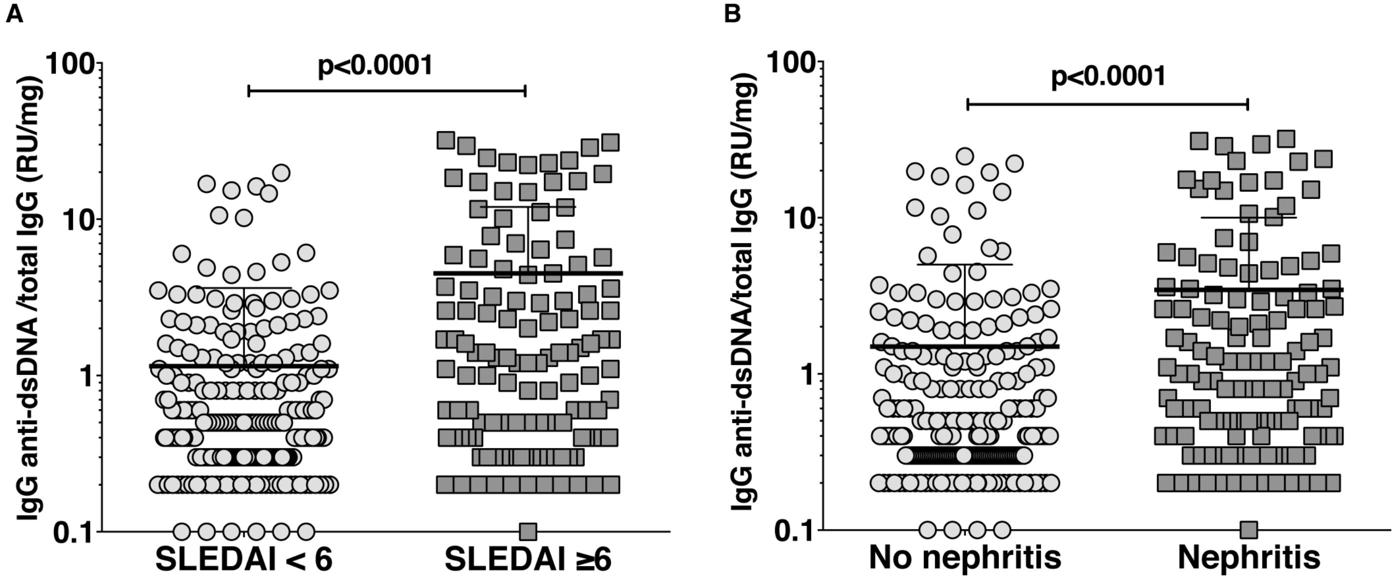

Figure S5. Serum IgG anti-dsDNA levels are higher in patients with high SLEDAI and nephritis

Serum IgG anti-dsDNA levels were measured in 396 SLE patients from the Swedish Karolinska cohort using multiplex bead analysis (BioRad, Bioplex 2200 ANA screen) and normalized for total IgG. **A.** The levels were significantly higher in patients with active disease determined by SLEDAI≥6 than patients with less active disease SLEDAI<6 (4.5±7.5 RU/ml n=126 vs 1.2±2.5 RU/ml n=270). Note that the SLEDAI-2K score includes a dsDNA component. **B.** The levels of IgG anti-dsDNA/total IgG were significantly higher in patients with a history of nephritis compared to patients without nephritis (3.5±6.6 RU/ml n=148 vs 1.5±3.5 RU/ml n=241). The nephritis group included both patients with active nephritis and patients in remission but with a previous history of nephritis. P-values are presented from 2-sided Mann-Whitney analysis.

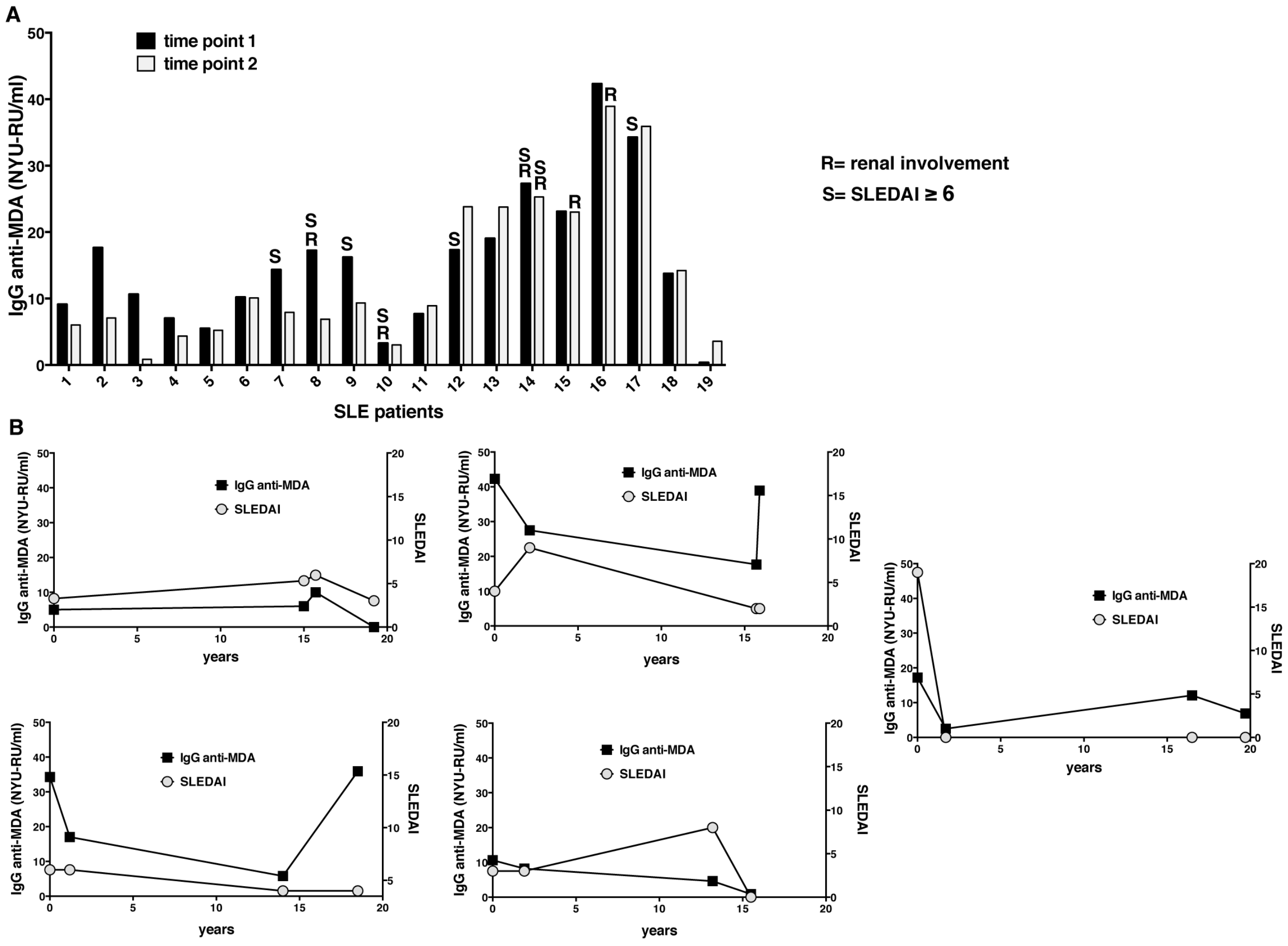

Figure S6. IgG anti-MDA level variation over long time

IgG anti-MDA levels were measured by ELISA and compared in 19 SLE patients that had cryopreserved biobanked serum samples with 11-20 years between visits. **A.** IgG anti-MDA levels for individual patients for first and last available time points. The average time between the time points were 17.8 years (11-20 years). If the patient had a renal involvement at the time of visit the bar is marked with **R** and if the patient had active disease SLEDAI≥6 the bar is marked with **S**. **B.** Variations of IgG anti-MDA levels in five patients that had samples available for several time points stretching over 20 years. SELENA-SLEDAI scores at the time of visit are depicted in the same graphs on the right y-axis.

Table S1. Correlation of oxidation-associated IgG autoantibody reactivity to PC and MDA with SLE-associated autoantibody reactivities

	**IgG anti-PC**			**IgG anti-MDA**		
	N	***Spearman R***	***P-value***	***N***	***Spearman R***	***P-value***
Total IgG	397	0.18	**0.0003**	397	0.46	**<0.0001**
IgG anti-dsDNA	398	0.00	NS (0.96)	398	0.42	**<0.0001**
IgG anti-nucleosome	398	0.07	NS (0.18)	398	0.38	**<0.0001**
IgG anti-ribosome	397	0.01	NS (0.91)	397	0.12	**0.02**
IgG anti-CL	324	0.19	**0.0006**	324	0.26	**<0.0001**
IgG anti-β2GPI	324	0.20	**0.0003**	324	0.26	**<0.0001**
IgG anti-Sm	398	0.11	**0.03**	398	0.18	**0.0004**
IgG anti-RNP-A	398	0.08	NS (0.09)	398	0.16	**0.002**
IgG anti-SSA/Ro52	398	-0.06	NS (0.23)	398	0.10	**0.04**
IgG anti- SSA/Ro60	398	-0.06	NS (0.21)	398	0.13	**0.01**
IgG anti-SSB/La	398	-0.12	**0.02**	398	-0.02	NS (0.76)

Statistical correlations were determined with Spearman analysis in 398 SLE patients from the Karolinska cohort.

Table S2. Association of IgG anti-dsDNA with serological and clinical measurements of disease

		**IgG anti-dsDNA/total IgG**	
	n	***Spearman R***	***p-value***
Demographics			
Age	397	-0.24	**<0.0001**
Disease duration	388	-0.23	**<0.0001**
Disease activity and damage indices			
SLEDAI	396	0.37	**<0.0001**
SLAM	396	0.14	**0.005**
SLICC	396	-0.10	NS (0.05)
Markers of kidney function			
Cystatin C eGFR	283	-0.08	NS (0.16)
U-albumin	373	0.35	**<0.0001**
Serum creatinine	395	-0.03	NS (0.55)
Complement factors			
C1q	361	-0.28	**<0.0001**
C2	304	-0.37	**<0.0001**
C3	385	-0.33	**<0.0001**
C4	385	-0.34	**<0.0001**
Inflammation biomarkers			
ESR	376	0.00	NS (0.94)
hsCRP	393	0.11	**0.03**
sTNFR-1	286	0.11	NS (0.07)
sTNFR-2	287	0.19	**0.001**
VCAM-1	283	0.18	**0.003**

Statistical correlations were determined with Spearman analysis in 397 SLE patients.

Table S3. Association of IgG anti-MDA with serological and clinical measurements of disease

		IgG anti-PC / total IgG		IgG anti-MDA / total IgG	
	N	***Spearman R***	***p-value***	***Spearman R***	***p-value***
Demographics					
Age	397	-0.17	**0.0006**	-0.07	NS (0.14)
Disease duration	388	0.03	NS (0.53)	-0.16	**0.001**
Disease activity and damage indices					
SLEDAI	396	-0.04	NS (0.38)	0.31	**<0.0001**
SLAM	396	-0.12	**0.02**	0.18	**0.0004**
SLICC	396	-0.15	**0.003**	-0.01	NS (0.77)
Markers of kidney function					
Cystatin C GFR	283	0.24	**<0.0001**	-0.20	**0.0008**
U-albumin	373	-0.06	NS (0.26)	0.23	**<0.0001**
Serum creatinine	394	0.008	NS (0.77)	-0.04	NS (0.41)
Complement factors					
C1q	361	-0.01	NS (0.87)	-0.18	**0.0008**
C2	304	-0.17	**0.003**	-0.24	**<0.0001**
C3	385	0.02	NS (0.66)	-0.24	**<0.0001**
C4	385	0.06	NS (0.27)	-0.24	**<0.0001**
Inflammation biomarkers					
ESR	376	-0.23	**<0.0001**	0.22	**<0.0001**
hsCRP	393	-0.27	**<0.0001**	0.17	**0.001**
sTNFR-1	286	-0.18	**0.003**	0.21	**0.0003**
sTNFR-2	287	-0.23	**0.0001**	0.35	**<0.0001**
VCAM-1	283	-0.22	**0.0002**	0.27	**<0.0001**

Statistical correlations were determined with Spearman analysis in 397 SLE patients from the Karolinska cohort.

Table S4. Correlation of antibodies and biomarkers with SLE disease and damage indices

		SLAM		SLEDAI		SLICC	
	N	***R-value***	***p-value***	***R-value***	***p-value***	***R-value***	***p-value***
Demographics							
Age	396	**-0.11**	**0.02**	**-0.23**	**<0.0001**	**0.48**	**<0.0001**
Disease duration	388	**-0.26**	**<0.0001**	**-0.23**	**<0.0001**	**0.36**	**<0.0001**
Oxidation-associated Abs							
IgG anti-PC	396	-0.04	0.42	0.03	0.52	**-0.20**	**<0.0001**
IgG anti-MDA	396	**0.24**	**<0.0001**	**0.34**	**<0.0001**	-0.08	0.12
SLE-associated Abs							
IgG anti-dsDNA	396	**0.20**	**<0.0001**	**0.46**	**<0.0001**	**-0.20**	**<0.0001**
IgG anti-nucleosome	396	**0.26**	**<0.0001**	**0.42**	**<0.0001**	**-0.20**	**<0.0001**
IgG anti-ribosome	396	0.06	0.22	**0.14**	**0.004**	-0.06	0.24
IgG anti-CL	323	0.02	0.78	**0.14**	**0.01**	0.10	0.08
IgG anti-β2GPI	323	0.00	1	**0.12**	**0.04**	0.06	0.27
IgG anti-Sm	396	**0.19**	**0.0001**	**0.22**	**<0.0001**	**-0.16**	**0.002**
IgG anti-Sm/RNP	396	**0.17**	**0.0005**	**0.19**	**0.0002**	-0.09	0.062
IgG anti-RNP-A	396	**0.13**	**0.009**	**0.17**	**0.0009**	**-0.12**	**0.02**
IgG anti-RNP-68	396	**0.11**	**0.03**	**0.13**	**0.01**	-0.07	0.14
IgG anti-SSA/Ro52	396	**0.10**	**0.046**	0.03	0.57	-0.06	0.24
IgG anti-SSA/Ro60	396	0.09	0.067	0.02	0.64	-0.05	0.36
IgG anti-SSB/La	396	0.04	0.41	-0.03	0.51	-0.07	0.17
Total IgG	396	**0.20**	**<0.0001**	**0.17**	**0.0008**	**-0.14**	**0.007**
Complement factors							
C1q	361	**-0.12**	**0.03**	**-0.27**	**<0.0001**	**0.15**	**0.006**
C2	304	0.02	0.75	**-0.24**	**<0.0001**	**0.14**	**0.01**
C3	384	-0.08	0.099	**-0.32**	**<0.0001**	**0.14**	**0.006**
C4	384	-0.10	0.054	**-0.35**	**<0.0001**	**0.14**	**0.005**
Inflammation biomarkers							
ESR	377	**0.46**	**<0.0001**	**0.24**	**<0.0001**	**0.13**	**0.01**
hsCRP	394	**0.16**	**0.001**	**0.11**	**0.03**	**0.20**	**<0.0001**
sTNFR-1	286	**0.19**	**0.001**	**0.13**	**0.03**	**0.31**	**<0.0001**
sTNFR-2	287	**0.28**	**<0.0001**	**0.31**	**<0.0001**	**0.26**	**<0.0001**
VCAM-1	283	**0.23**	**0.0001**	**0.26**	**<0.0001**	**0.12**	**0.04**

Statistical correlations were determined with Spearman analysis in 396 SLE patients from the Karolinska cohort.

Table S5. Clinical characteristics of SLE patients with high IgG anti-MDA/total IgG measurements

	**Low IgG anti-MDA/total IgG#** *(N=270)*	**High IgG anti-MDA/total IgG#** *(N=126)*		
	Frequency (% n/N) ***Mean*± SD (N)**	***Frequency (% n/N)*** **Mean ± SD (N)**	***Odds Ratio [95% CI]***	***p-value€***
Age (years)	47±15 (270)	46±15 (126)	N/A	NS (0.81)
Disease duration (years)	13.9±12 (270)	11.7±13 (126)	N/A	**0.008**
Female	91% (245/270)	84% (106/126)	0.54 [0.29-1.02]	NS (0.06)
Current prednisolone (mg/d)	5.8±8.8	7.2±10.6	N/A	NS (0.28)
ESR (mm/h)	23.4±18.7 (256)	32.1±23.7 (120)	N/A	**0.0002**
U-albumin	75±292 (255)	382±1380 (117)	N/A	**0.0005**
ANA positivity*	85% (167/197)	90% (75/83)	1.68 [0.74-3.85]	NS (0.25*)*
IgG anti-dsDNA positivity*	28% (76/269)	58% (73/126)	3.50 [2.25-5.44]	**<0.0001**
APS autoantibody profile**	19% (45/232)	32% (33/103)	1.96 [1.16-3.31]	**0.02**
SS autoantibody profile**	31% (71/232)	26% (27/103)	0.81 [0.48-1.36)	NS (0.44)
SLICC >1	37% (100/270)	36% (45/125)	0.96 [0.61-1.49)	NS (0.91)
SLAM >6	44% (120/270)	56% (70/126)	1.56 [1.02-2.39]	**0.04**
SLEDAI≥6	25% (66/268)	46% (58/125)	2.65 [1.69-4.15]	**<0.0001**
Butterfly rash	53% (142/266)	38% (47/124)	0.53 [0.35-0.82]	**0.005**
Discoid cutaneous	22% (59/266)	12% (15/124)	0.48 [0.26-0.89]	**0.02**
Photosensitivity	70% (186/266)	56% (70/124)	0.56 [0.36-0.87]	**0.01**
Oral ulcer	36% (97/266)	23% (29/124)	0.53 [0.323-0.86]	**0.01**
Arthritis	81% (216/266)	86% (107/124)	1.45 [0.80-2.65]	NS (0.25)
Serositis	37% (97/165)	43% (53/124)	1.29 [0.94-2.0]	NS (0.26)
Neurological manifestations	11% (28/265)	11% (13/124)	0.99 [0.49-1.99]	NS (1)
Leukocytopenia	50% (132/265)	47% (58/124)	0.89 [0.58-1.36]	NS (0.59)
Lymphocytopenia	48% (128/264)	56% (69/124)	1.33 [0.87-2.05]	NS (0.19)
Thrombocytopenia	18% (46/264)	23% (28/122)	1.41 [0.83-2.39]	NS (0.21)
Nephritis	32% (86/265)	50% (62/124)	2.14 [1.35-3.22]	**0.001**
Arterial events	13% (33/263)	19% (23/212)	1.63 [0.91-2.93]	NS (0.12)
VTE	13% (35/262)	16% (20/122)	1.27 [0.70-2.31]	NS (0.44)
Vascular events§	22% (58/260)	33% (40/120)	1.74 [1.08-2.81]	**0.03**

n= Number of positive patients; N= Total number of patients with available data;
VTE= venous thromboembolism, includes events of deep vein thrombosis and pulmonary embolism.
§History of vascular events, includes both arterial and venous events
Cut-off for IgG anti-MDA/total IgG was based on the highest quartile, 75th percentile of controls (>3.8 KI-RU/mg)
€ p-values from Fisher's exact test or Mann-Whitney analysis
* Positive at the time of sample collection.
** Antiphospholipid syndrome (APS) vs Sjögren's syndrome (SS) autoantibody profiles based on definitions in Grönwall et al Clin Immunol. 2017. APS-profile: two or more IgG/IgA/IgM anti-CL/β_2GPI positive tests, and/or a lupus anticoagulant (LA) positive test; SS-profile: two or more positive IgG anti-Ro52/Ro60/La tests.
